# Multiple cytogenetics forms of *KMT2A* amplification and jumping translocation in a case of myelodysplastic syndrome

**DOI:** 10.1002/jha2.313

**Published:** 2021-10-12

**Authors:** Antoine Ittel, Norbert Vey, Marie‐Joëlle Mozziconacci

**Affiliations:** ^1^ Biopathology Department Centre de Recherche en Cancérologie de Marseille (CRCM) Institut Paoli‐Calmettes Marseille 13009 France; ^2^ Haematology Department, CRCM Institut Paoli‐Calmettes Marseille 13009 France

**FIGURE 1:**
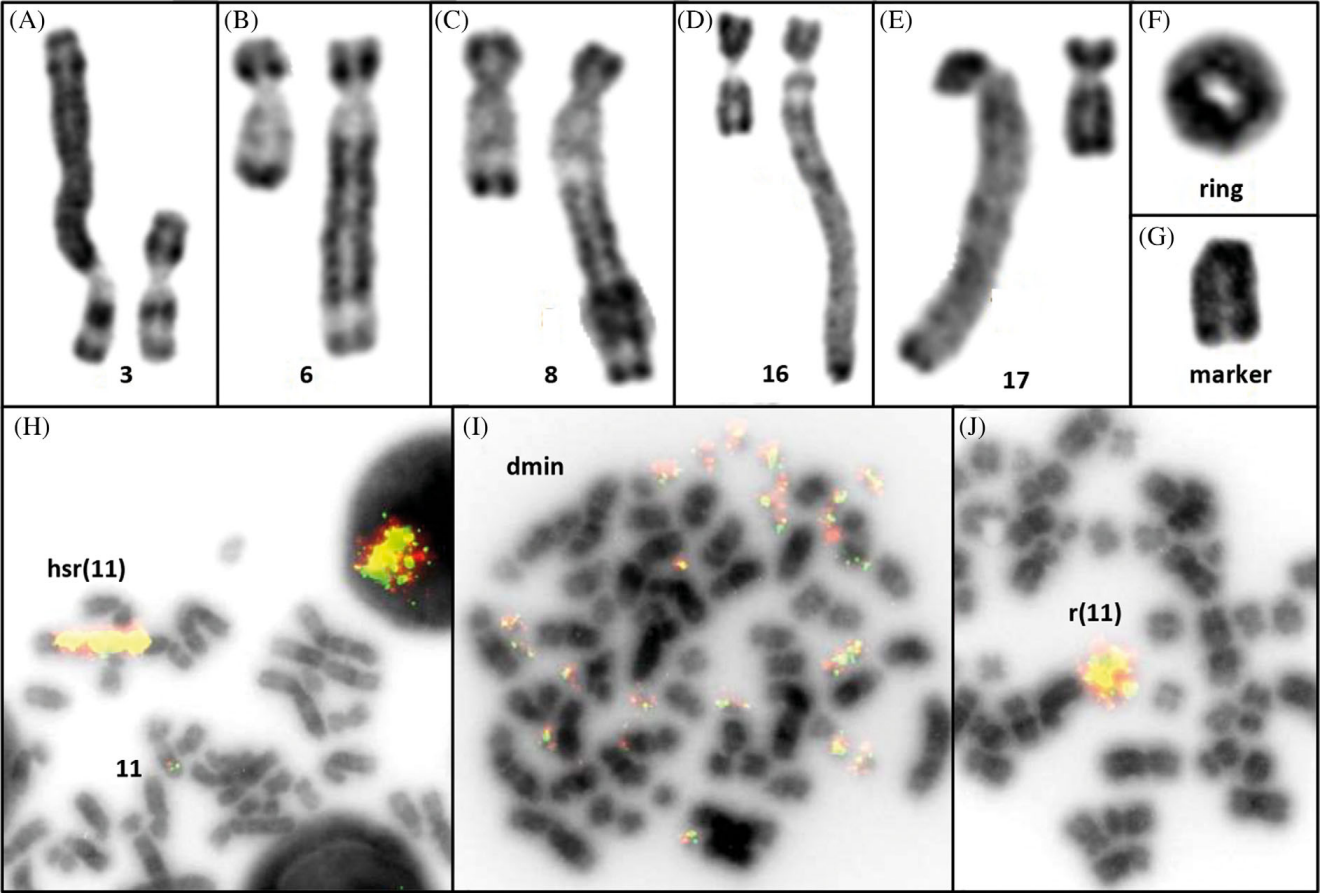
KMT2A amplification as an hsr on different chromosomes by jumping translocation (A to E and H), a ring (F and J), a marker chromosome (G) or double minutes (I). RHG banding and KMT2A breakapart probe FISH (Vysis, Abbott Molecular)

In 2012, a 77‐year‐old woman was diagnosed with low‐risk myelodysplastic syndrome (MDS). In 2017, 5 years after diagnosis, her blood count showed anemia (96 g/l) and a medullar examination showed multi‐lineage dysplasia with 8% blast cells. Cytogenetic analysis on bone marrow cells showed an isolated 5q deletion. An erythropoietin treatment was started with initially a major response. In 2019, 7 years after diagnosis, in front of the anemia aggravation, lenalidomide was initiated and stopped 1 year later because of pulmonary hypertension. A growth‐factor‐based‐treatment was initiated, without effect. In June 2021, the bone marrow examination showed 16% blast cells and the medullar karyotype (RHG banding, Figure 1A) still showed the 5q (Figure 1A) deletion with the occurrence of an unexpected cytogenetic amplification taking several forms: jumping *hsr* (homogeneously staining region) inserted in different chromosomes: short arm of chromosome 3 (Figure 1A) and long arm of chromosomes 6 (Figure 1B, H), 8 (Figure 1C), 16 (Figure 1D) or 17 (Figure 1E); an additional ring chromosome (Figure 1F, J) or additional marker chromosome (Figure 1G). A *KMT2A* amplification was detected by fluorescence in situ hybridization (*FISH*, Vysis LSI MLL Dual Color, Break Apart Rearrangement Probe Abbott Molecular) and confirmed karyotype findings. In July 2021, the MDS transformed in acute myeloid leukemia (AML) with 21% medullar blast cells and the same cytogenetic abnormalities. A treatment based on venetoclax and azacytidine was then initiated. The patient was still alive in September 2021.

*KMT2A* amplification is a rare cytogenetic event (<1%) mostly seen in AML and MDS, traditionally associated with an aggressive clinical course, poor response to chemotherapy and extremely short survival. The distinctive characteristic of our case lies in the presence of all variable cytogenetic manifestations known in amplification such as *hsr*, ring chromosomes and double minute chromosomes (the latter was only seen with FISH, Figure 1), in a sole case. It is also interesting to note that the *hsr* is present on different chromosomes as a jumping translocation, another rare cytogenetics event.

